# Social Media Versus Customized Mobile Apps for Online Perinatal Mental Health Support: The Potential for a Hybrid Model

**DOI:** 10.2196/107553

**Published:** 2026-08-31

**Authors:** Angela G Campbell

**Affiliations:** 1 Department of Applied Health Science, School of Public Health Bloomington, Indiana University Bloomington, 1025 E 7th St, Bloomington, IN, 47401, United States, 1 (812) 855-1561

**Keywords:** perinatal mental health, maternal mental health, social media, mobile apps, digital mental health, rural health, postpartum mental health, postpartum depression, postpartum anxiety

## Abstract

A recent participatory co-design study for a social media–based mental health intervention targeting perinatal women in regional, rural, and remote communities identified five core intervention needs: peer support and connection, personalized care, access to information that is trustworthy and eases uncertainty, place and culturally specific support, and a digital platform that is accessible and easy to use. This commentary argues that digital platform selection should be addressed as an active intervention component. A hybrid model that uses the built-in user base and accessibility of social media alongside the increased privacy and content control afforded by a customized mobile app could maximize the benefits of both platforms to more fully address all components of the proposed intervention.

## Platform Choice Is an Integral Part of an Online Intervention

Lynch et al [1] identified five needs for social media interventions among perinatal women in Northern Queensland: connection and support from peers; health care that is tailored to personal needs; access to medical information that eases uncertainty; support that acknowledges location and culturally specific needs; and accessible, user-friendly digital platforms to access content. The study appropriately treats social media as a set of benefits and constraints rather than a neutral means of accessing content. The next phase of this work could extend this insight by treating the choice of digital platform as an intervention component that requires evaluation for optimized implementation. Social media was used for this study, but customized mobile apps are also being developed for digital mental health interventions and could address similar needs but with a distinct array of pros and cons [2]. Moreover, the choice of platform for an online intervention does not have to be a dichotomous choice between social media and a customized mobile app. A hybrid approach could maximize the positive aspects of both platforms and allow for maximum reach while optimizing content and user experience.

## Existing Social Media Platforms: Reach With Limited Control

Existing social media platforms offer advantages that are difficult to reproduce in custom-built applications. They have large user bases, familiar interfaces, low onboarding burden, and tools for discovering local or interest-based communities. For perinatal populations in regional, rural, and remote settings, these features may facilitate peer support and location-based connection without requiring users to adopt an unfamiliar platform.

While social media has the potential to facilitate access to mental health support, social media can also be addictive [3], damage mental health by disseminating misinformation [4], encourage upward comparisons of idealized motherhood experiences [5], and increase anxiety and poor body image due to comparisons with other postpartum individuals [6]. These hazards must be understood as part of the intervention context because the positive aspects of the intervention cannot be isolated from the delivery platforms. The potential harms should be actively weighed against the benefits of the platform when choosing how the intervention is delivered to participants. Strategies to guard against these exposures should be actively developed and incorporated as part of the intervention implementation.

## Customized Mobile Apps: Control With Adoption Challenges

Customized mobile apps can provide a more controlled digital environment relative to social media interest groups. Developers can curate evidence-based content; restrict advertising; implement consent and privacy controls; tailor materials by perinatal stage or location; and embed screening, referral, and accessibility features. They can also reduce exposure to unrelated content that may undermine the intended intervention, such as fitness-related posts that could exacerbate postpartum body image issues [6].

Nevertheless, greater control does not always correlate with greater effectiveness. Evidence for app-based perinatal mental health interventions is mixed, and benefits depend on content, usability, engagement, and implementation support [7,8]. A new app must earn trust, overcome download and registration friction, and sustain use during the perinatal period, which is a limited time in a person’s life with multiple competing demands for attention. Community functions are particularly difficult to establish because location-based groups divide a relatively small population into even smaller cohorts. Integration with health care systems, insurers, or trusted community organizations may improve reach, but this requires empirical testing [8]. Customized mobile app platforms also need durable plans for funding, software maintenance, moderation, and data stewardship, as a platform that becomes obsolete or is unsupported is not a long-term solution. This type of funding may require advertising revenue or fees for users, which could also deter the adoption and prolonged use of the app. Thus, the customized app maximizes content control, but this may come at the cost of reach and community building, which has been highlighted as a central need for the intervention [1].

## Considering a Hybrid Model of Platform Use

A promising architecture for perinatal mental health interventions may be a hybrid model that uses social media as a funnel into a customized app. This approach has shown some promise in postpartum populations relative to controls for improving breastfeeding confidence and perceived social support [9]. A perinatal mental health intervention could adapt the structure of this type of intervention for targeted mental health outreach and support.

The strength of social media lies in the existing user base and ability to perform outreach to specialized populations. Social media platforms are ideal for recruitment to an intervention that exists as a separate customized mobile app. The customized app could be downloaded via a link in the social media outreach advertisements and would provide a more controlled environment for the delivery of tailored modules, private peer interaction, screening, referrals, and crisis support. The transition between environments should be effortless and transparent about who controls each space and how user data are handled, as privacy and trust are often a strong consideration for users when sharing personal information [10]. Assigning specific domains to each platform in the intervention design and evaluating their effectiveness would make the platform’s contribution to the success of the intervention visible rather than attributing all outcomes to the intervention content.

## Conclusions

Digital interventions have the potential to serve as low-cost, first-line tools for outreach and prevention among difficult to reach populations, such as those in rural areas or other low-resource settings. Lynch et al [1] make an important contribution by assessing the community needs for a social media intervention in an underserved population. The next design question may be an assessment of whether social media is the best platform for the intervention or if the strengths of social media can be combined with a more customized mobile app platform to meet user needs. Treating platform architecture as an active and measurable component of the intervention will strengthen safety, interpretability, and sustainability for implementation in future trials. It will also help researchers measure what aspects of the intervention effectiveness are due to the intervention content and which ones are due to the delivery platform.
